# A qualitative study into the personal factors influencing secondary school teachers’ motivating styles

**DOI:** 10.3389/fpsyg.2023.1127090

**Published:** 2023-07-20

**Authors:** Woon Chia Liu, Leng Chee Kong, Chee Keng John Wang, Ying Hwa Kee, Betsy Ng, Karen Lam, Johnmarshall Reeve

**Affiliations:** ^1^National Institute of Education, Nanyang Technological University, Singapore, Singapore; ^2^Curriculum Policy Office, Ministry of Education, Singapore, Singapore; ^3^Institute for Positive Psychology and Education, Australian Catholic University, Sydney, VIC, Australia

**Keywords:** self-determination theory, motivating styles, autonomy-supportive motivating style, controlling motivating style, personal factors, mathematics and science teachers

## Abstract

**Background:**

All teachers aspire to create the most motivating classroom climate for their students. This is because students who are motivated demonstrate superior learning outcomes relative to students who are not motivated. According to the Self-Determination Theory (SDT), when teachers establish an autonomy-supportive climate in their classrooms, their students can benefit in numerous developmentally and educationally important ways. Whilst most teachers have an understanding that autonomy-supportive teaching can benefit their students, many of them are unwilling or unable to carry out autonomy-supportive strategies. This can be explained by the implicit and explicit forces (e.g., teaching philosophies and beliefs, personal experiences) imposed on them by their internal (namely, intrapsychic) and external (namely, social) environments. This paper focuses on the personal factors that influence teachers’ adoption and employment of autonomy-supportive instructional behaviours.

**Method:**

Following a 10-week intervention program on implementing six autonomy-supportive instructional behaviours, we interviewed 59 teachers from 17 secondary schools in Singapore on their adoption and employment of the teaching strategies. Their number of years of teaching experience ranged from 1 to 31 years with a mean of 10.8 years of teaching experience, and 62.71% of them were female.

**Finding:**

From the interviews, we identified several teacher-related personal factors which we labelled “teaching philosophies and beliefs,” “personal experiences,” “motivation to teach,” “personality,” “teachers’ mental and emotional states” and “teaching efficacy.” Through identifying the personal factors, we hope to raise awareness amongst the teachers on the inner forces that can foster or frustrate their own expression of autonomy-supportive instructional behaviours. Given the many plausible benefits that can be derived from autonomy-supportive teaching, we hope that the information gained from this qualitative study can path the way for greater willingness and effort in implementing autonomy-supportive teaching in the classrooms.

## 1. Introduction

All teachers aspire to create the most motivating classroom climate for their students. This is because students who are motivated demonstrate superior developmental and educational learning outcomes, relative to students who are not motivated (Flunger et al., 2019; Abula et al., 2020; Barkoukis et al., 2020). The Self-Determination Theory (SDT; Deci and Ryan, 2000) presents a framework for understanding motivated behaviours and in nurturing a classroom climate that is motivating. In this paper, we will understand motivation from the perspective of the SDT and investigate some personal factors that may facilitate the teachers in creating a classroom environment that is motivating.

### 1.1. Self-determination theory

The SDT conceptualised motivation ─ amotivation, external regulation, introjected regulation, identified regulation and intrinsic regulation as occurring in a spectrum with increasing level of autonomy. In SDT conceptualisation, amotivation is least adaptive because an individual who is amotivated lacks motivation to act. Along the spectrum, external and introjected regulations represent controlled regulations in that externally regulated behaviours are controlled by external means such as rewards and punishments and introjectedly regulated behaviours are controlled by internal means such as guilt or the desire to protect or boost the ego. Identified and intrinsic regulations represent the more autonomous regulations in that both regulations emanate from the individual’s own values and choices, respectively. Amongst these regulations, intrinsic regulation is deemed the most adaptive form of regulation and one in which SDT theorists advised to nurture in our students (Vallerand et al., 1992; Deci and Ryan, 2000).

Within the macro SDT are two mini theories ─ the Organismic Integration Theory (OIT) and Cognitive Evaluative Theory (CET) (Deci and Ryan, 2000) which detail the processes of motivation and what educators can do to foster the more autonomous regulations in learning. According to the OIT, how autonomous the motivational regulation is depends on how much the student has internalised (grasps the underlying meaning and value of) and integrated (transforms) the regulation into his/her existing sense of self (Ryan and Deci, 2000). Internalisation thus represents the interpersonal exchange between the student and his/her social environment and integration represents the intrapersonal transformation that occurs within the student. In the CET, SDT theorists (Ryan et al., 1985; Ryan and Deci, 2000) proposed that the social environment can facilitate or hinder the processes of internalisation and integration. In the domain of education and within the social context of the classroom, one factor that may foster or frustrate the internalisation and integration processes is the teachers’ motivating styles.

### 1.2. Motivating styles

Undeniably, teachers wield great influence over the classroom climate and what they say and do can affect their students’ motivation in learning. SDT theorists observed that what a teacher says and does in a classroom can take on a recurring and enduring pattern, and they termed this persisting pattern the teacher’s motivating style (Deci et al., 1981; Reeve, 2009, 2016). Reeve (2009, 2016) spoke of two types of motivating styles ─ autonomy-supportive and controlling styles. He defined an autonomy-supportive teaching style as being characterised by an interpersonal tone that appreciates, nurtures, and vitalises students’ inner motivational resources and it can be demonstrated through instructional behaviours such as adopting the students’ perspective, acknowledging and accepting expressions of negative affect, and providing explanatory rationales (Reeve, 2009, 2016; Reeve and Shin, 2020). SDT researchers further explained that in an autonomy-supportive climate, teacher-student interactions tend to be reciprocal and flexible (Assor et al., 2002, 2005; Reeve et al., 2004; Reeve and Jang, 2006; Reeve, 2009, 2016). As its theoretical opposite, the controlling style is characterised by an interpersonal tone of pressure that students should think, feel, perform, and behave in a teacher-determined way and controlling instructional behaviours can manifest in the forms of directives, commands, and pressuring language or the offering of extrinsic rewards for desired student behaviours (Reeve, 2009, 2016). In a controlling climate, the teacher-student interactions tended to be top-down and uni-directional (Assor et al., 2002, 2005; Reeve et al., 2004; Reeve and Jang, 2006; Reeve, 2009, 2016). Table 1 provides the definition, enabling conditions and instructional behaviours associated with each motivating style (Reeve, 2009, 2016).

**Table 1:** Definitions, enabling conditions, and instructional behaviours associated with autonomy-supportive and controlling styles.

	Autonomy-support	Control
Definition	Interpersonal sentiments and behaviours teachers provide during instructions to identify, nurture, and develop students’ inner motivational resources.	Interpersonal sentiments and behaviours teachers provide during instructions to pressure students to think, feel, or behave in a specific way.
Enabling conditions	•Adopt the students’ perspective. •Welcome students’ thoughts, feelings, and actions. •Support students’ motivational development and capacity for autonomous self-regulation.	•Adopt the teacher’s perspective. •Intrude into students’ thoughts, feelings, or actions. •Pressure students to think, feel, or behave in a specific way.
Instructional behaviours	•Take the students’ perspective •Vitalise inner motivational resources •Provide explanatory rationales •Use non-pressuring informational language •Acknowledge and accept negative affect •Display patience	•Adopt only the teacher’s perspective •Use environmental sources of motivation •Give directives without explanations •Rely on pressuring language •Counter and try to change expressions of resistance and negative affect •Impatiently intrude into student’s workspace

### 1.3. Benefits of autonomy-support and costs of control

A growing body of research has offered evidence for the benefits of autonomy-support and the costs of control. The numerous benefits of autonomy-support include students having more autonomous motivation and less amotivation (Black and Deci, 2000; Vansteenkiste et al., 2004; Soenens and Vansteenkiste, 2005; Cheon and Reeve, 2013; Cheon and Reeve, 2015; Ng et al., 2015; Wang et al., 2016; Fin et al., 2019; Abula et al., 2020; Barkoukis et al., 2020), greater psychological well-being (Black and Deci, 2000; Assor et al., 2002; Kaplan and Assor, 2012; Flunger et al., 2019), greater classroom engagement (Assor et al., 2002; Cheon and Reeve, 2013; Cheon and Reeve, 2015; De Meyer et al., 2016; Jang et al., 2016; Michou et al., 2023), greater persistence (Vansteenkiste et al., 2004), deeper conceptual learning (Vansteenkiste et al., 2004), better achievement (Black and Deci, 2000; Vansteenkiste et al., 2004; Soenens and Vansteenkiste, 2005; Wang et al., 2016), and more prosocial behaviours and less behavioural problems (Kaplan and Assor, 2012; De Meyer et al., 2016; Assor et al., 2018; Cheon et al., 2019). The costs of control include students reporting greater amotivation (Cheon and Reeve, 2015), more frequent experience of negative emotions (Assor et al., 2002), less classroom engagement (Assor et al., 2002; Cheon and Reeve, 2015; De Meyer et al., 2016; Jang et al., 2016), and more behavioural problems (De Meyer et al., 2016). Whilst students can benefit from their teachers’ autonomy-support, autonomy-supportive teaching can be rewarding for the teachers as well with teachers reporting greater teaching motivation, teaching efficacy and better teaching well-being such as greater vitality, job satisfaction and lesser emotional and physical exhaustion, and better relationship with students (Cheon et al., 2014, 2020).

SDT theorists supposed and empirical information from SDT-based interventions showed that it is possible for teachers to learn to be autonomy-supportive, and this autonomy-supportive teaching can benefit both the students and the teachers (Reeve and Cheon, 2014, 2016, 2021). Despite the numerous potential benefits, some teachers are not willing to or are not able to adopt the autonomy-supportive style and if they do, are not consistent in their practices. Past studies suggest that several factors that are school-related, teacher-related and student-related can influence the teachers towards a more autonomy-supportive or a more controlling motivating style (Pelletier et al., 2002; Reeve, 2009). In this paper, we will focus on understanding the teacher-related factors or the teachers’ personal factors that influence their willingness and ableness in adopting and implementing autonomy-supportive teaching.

### 1.4. Teacher-related factors influencing the teachers’ motivating style

Specific to the teacher-related factors, past research suggested that the teachers’ psychological needs satisfaction (Taylor et al., 2008; Moè and Katz, 2021; Vermote et al., 2022), autonomous motivation (Pelletier et al., 2002; Taylor et al., 2008; Hein et al., 2012), personal dispositions such as autonomous causality orientation and self-compassion (Deci and Ryan, 1985; Taylor et al., 2008; Reeve et al., 2018; Moè and Katz, 2020), intrinsic value to teach (Parr et al., 2021), teaching ability and efficacy (Leroy et al., 2007; Bennett et al., 2017; Parr et al., 2021), and teachers’ beliefs (Reeve and Cheon, 2014) can influence the teachers’ adoption of autonomy-supportive teaching.

For example, Taylor et al. (2008) found that when teachers’ needs were fulfilled, they were more likely to try to gain an understanding of their students, provide them with help, and provide meaningful rationale and choice to the students. Likewise, teachers with autonomous motivation were found to be more likely to be student-centred or utilise productive teaching styles, whereas teachers with non-autonomous motivation were found to be more teacher-centred or utilise reproductive teaching styles (Pelletier et al., 2002; Taylor et al., 2008; Hein et al., 2012; Solstad et al., 2015). In other words, the more self-determined teachers feel, the more they are likely to create a self-determined learning climate for students. Additionally, teachers’ personal disposition (Taylor et al., 2008) for example autonomous causality orientation (Deci and Ryan, 1985) and self-compassion (Moè and Katz, 2020), play a role in predicting teachers’ use of motivational strategies. And when the teachers had intrinsic value (having an internal desire or passion) in teaching, they intuitively would prioritise their students’ needs and interests. Also found previously was that the teachers’ perceived teaching ability, that is, their confidence or teaching efficacy determined their willingness and ability to experiment with pedagogies such as in providing more choices, allowing more criticism, and fostering more understanding (Parr et al., 2021). Finally, when teachers believed that autonomy-supportive strategies were culturally normal and effective, they were more likely to implement the motivating style, suggesting that teacher beliefs can predict teachers’ motivating styles (Reeve et al., 2014). And this leads us to the purpose of the study and the research question.

### 1.5. Purpose of study

Whilst there are studies on the outcomes of autonomy-supportive teaching, few had focussed on the antecedents of adopting this approach and fewer had examined the personal factors influencing this practice from a qualitative perspective. This study aimed to fill in the gap by identifying the personal factors and qualitative reasons that influence the teachers’ adoption and employment of autonomy-supportive instructional behaviours. Through identifying the personal factors, we hope to raise awareness amongst the teachers on the implicit and explicit inner forces that can foster or frustrate their own expression of autonomy-supportive teaching. We also hope to raise awareness amongst the people such as the policy makers and school leaders who support them, on the personal factors influencing the teachers’ adoption and implementation of autonomy-supportive teaching strategies. Since the personal reasons reside in the minds of the teachers and they are the ones most privy to their own thoughts and experiences, the only way to better understand and support them is to first get them to articulate their thoughts and experiences.

Given the many plausible benefits (Black and Deci, 2000; Assor et al., 2002; Vansteenkiste et al., 2004; Soenens and Vansteenkiste, 2005; Kaplan and Assor, 2012; Cheon and Reeve, 2013; Ng et al., 2015; De Meyer et al., 2016; Jang et al., 2016; Wang et al., 2016; Assor et al., 2018; Cheon et al., 2019, 2020; Fin et al., 2019; Flunger et al., 2019; Abula et al., 2020; Barkoukis et al., 2020; Michou et al., 2023) that can be derived for both the teachers and students from autonomy-supportive teaching, we hope that the information gained from this qualitative study and the enhanced awareness that it accorded the teachers can path the way for greater willingness and effort in implementing autonomy-supportive teaching in the classrooms. We also hope the information acquired from the findings can pave the way for greater support for the teachers in creating a motivating climate in their classrooms.

Our overarching research question for this study is thus as follows: What personal factors influence teachers’ adoption of autonomy-supportive motivating style?

## 2. Methods

### 2.1. Research Design

This qualitative study was part of a larger experimental study which utilised an autonomy-supportive intervention as the experimental protocol. As background information, the larger experimental study involved a 2 (intervention versus control group) X 3 (pre, mid and post-intervention) experimental research design. The retrospective qualitative interviews (the findings of which are presented in this paper) on the teachers’ experiences in implementing the autonomy-supportive instructional strategies were conducted after the experimental teachers had attended the teacher training on autonomy-supportive instructional behaviours, attempted the strategies in their classrooms and saw through the completion of the intervention program.

#### 2.1.1. The autonomy-supportive intervention

The training programme that the teachers participated in was an amalgamation of the Autonomy-Supportive Classroom Intervention used by Wang and his colleagues in Singapore (Ng et al., 2015; Wang et al., 2016), and the Autonomy-Supportive Intervention Program used by Reeve and his colleagues (Reeve et al., 2004; Reeve and Cheon, 2014; Cheon and Reeve, 2015; Cheon et al., 2018).

There were three parts to the training session. First, the teachers were introduced to the principles of the SDT, autonomy-supportive versus controlling motivating style, six autonomy-supportive instructional behaviours contrasting with six controlling instructional behaviours and presented with the empirical evidence on the benefits of autonomy-support and the costs of control. Second, they practised on the “how to” of the six autonomy-supportive acts. Third, they convened after a month of exploring with autonomy-supportive teaching to share about their field experiences and the strategies that had worked for them. Table 2 presents an overview of the 3-part teacher training programme.

**Table 2:** Overview of the teacher training programme.

	Session	Activity
1.	Understanding the What, Why and How (3 h)	Activity 1: Use of vignettes to encourage teachers to reflect on their own motivating style (Reeve and Cheon, 2014) Activity 2: Discussion on the extent to which teachers are currently relying on controlling teaching strategies such as negative conditional regard, controlling rewards, intimidation and excessive personal control (Bartholomew et al., 2010) Activity 3: Present SDT tenets, autonomy-supportive and controlling motivating styles, provide empirical evidence on the benefits of autonomy support and the cost of control, and introduce the 6 instructional behaviours
2.	Practising the How (3 h)	Activity 1: Provide examples of the 6 instructional behaviours Activity 2: Role playing of the instructional behaviours Activity 3: Discussion on how realistic, how effective and how easy to implement autonomy-supportive instructional behaviours, as well as how to transform controlling behaviours
3.	Sharing tips and addressing concerns (2 h)	Activity 1: Recap of Session 1 and 2 Activity 2: Peer sharing of classroom experiences with autonomy supportive teaching and exchange of tips and strategies from the field

### 2.2. Participants

Fifty-nine mathematics and/or science teachers participated in the interviews. They were from 17 secondary schools randomly spread across the city-state of Singapore. Their mean number of years of teaching experience was 10.8 years, with teaching experiences ranging from 1 to 31 years, and 62.71% of them were female. These 59 teachers who were interviewed on their adoption and implementation of autonomy-supportive instructional behaviours were from the intervention group. Hence, they were purposively sampled within a random sampling frame.

### 2.3. Procedure

In accordance with the Declaration of Helsinki, we obtained approvals from our university’s Institutional Review Board and the Ministry of Education (Singapore) Corporate Research Office before inviting the schools to participate in the research study. Altogether, 17 schools responded to our invitation. Through the school key personnels, we acquired a list of teachers who could and were willing to participate in the intervention study.

To minimise possible teacher-related confounds, we randomly assigned the teachers who consented to the study into either the experimental or control group. The experimental teachers next attended the aforementioned training programme and then implemented the autonomy-supportive instructional behaviours in their classrooms for 10 weeks, whilst the control teachers did not attend the training programme and taught in their usual teaching style. At post-intervention, the experimental teachers were interviewed on their experiences with autonomy-supportive teaching. As the intention of the interviews was to identify the personal factors influencing the teachers’ adoption and implementation of autonomy-supportive motivating strategies, this purposive interview necessitated the teachers to have attended the teacher training, understood the tenets of autonomy-supportive motivating style, and attempted the autonomy-supportive instructional behaviours in their classrooms. Hence, only the experimental teachers were interviewed for this study. The interview questions are at attached Annex A.

### 2.4. Role of researcher, data collection, and data analysis

Throughout the intervention process, we (the researchers) played consultatory and observer roles. For the qualitative data collection, we assumed the role of “human instruments” (Greenbank, 2003) in conducting retrospective interviews with the teachers using a semi-structured interview protocol developed with reference to the work done by Reeve and his colleagues (Reeve et al., 2004; Reeve and Jang, 2006; Cheon et al., 2012; Cheon and Reeve, 2015; Reeve, 2016).

The 59 interviews, each lasting between 20 to 66 min, yielded a total of 2030 min of interview content. The interviews were audio-recorded, and the recordings transcribed. The transcripts were then manually analysed for themes related to teacher-related personal factors that had influenced the teachers’ willingness and ableness in conducting autonomy-supportive teaching. Following Braun and Clarke’s (2006) approach, the transcripts were repeatedly read for the identification of themes and for insightful responses. To improve the validity and credibility of the coding of the themes, they were cross-checked by two researchers (Braun and Clarke, 2006). Between the researchers, we initially identified 7 themes, and these were unanimously reduced to 6 themes after discussion. Specifically, the themes “Teaching Philosophies” and “Teaching Beliefs” were found to have considerable overlaps in meaning and excerpts extracted from the interviews. They were then combined to form one theme “Teaching philosophies and beliefs.” The themes were then reviewed by the principal investigator who agreed with the categorisation. Amongst the researchers, there was 100% inter-rater agreement on the themes identified.

We present the findings in the section below. In our presentation, each teacher was referred to by the abbreviation of his/her school, teacher code and gender.

## 3. Findings

In response to our research question “What personal factors influence teachers’ adoption of autonomy-supportive motivating style?,” we identified several themes which are summarised in Table 3. We will discuss the themes in turn.

**Table 3:** Teacher-related personal factors influencing the adoption and implementation of autonomy-supportive motivating style.

Theme	Frequency count
Teaching philosophies and beliefs	49 of 59 teachers
Personal experiences	32 of 59 teachers
Motivation to teach	14 of 59 teachers
Personality	13 of 59 teachers
Mental and emotional states	5 of 59 teachers
Teaching efficacy	2 of 59 teachers

### 3.1. Teaching philosophies and beliefs

From the interviews, we gathered that the degree and extent to which the teachers employed autonomy-supportive teaching was determined by their teaching philosophy, and their teaching philosophy guided by their beliefs (49 of 59 teachers) about teaching and learning. By “teaching philosophies and beliefs,” we refer to the teachers’ worldviews and perspectives about teaching and the roles of teachers. For the teachers who were more receptive of autonomy-supportive teaching, they were more likely to embrace the teaching philosophies of nurturing the child, the relationship, and the environment.

On nurturing the child, Teacher S9T61 (female) narrated on her teaching philosophy on “every child matters,” which guided her instructional behaviours. She shared, “I use encouraging words and that has always been the principle of me being a teacher. It’s really leave no student behind and never to let them feel that they are being threatened in an environment. I have seen teachers who are very threatening, who use very strong languages in class to inject fear into students and I have seen them (the students) breaking down as well, so to me, being autonomy-supportive in a way like the tone and the language and the body language being supportive and encouraging, that align with what I truly feel what a teacher should be, so that has always been the factor that has encouraged me.”

In nurturing the child, the teachers’ aims were for the students to be engaged in learning, to enjoy learning, to be motivated in learning and to be autonomous learners. Intrinsically, they were motivated to employ pedagogical moves that would conduce towards such learning outcomes. Teacher S15T101 (female) spoke of her belief and aim, “I believe that for students to want to learn, that love and curiosity for learning is very important. So in what I do, I try to find different ways to engage them to be curious and ask questions. So I strongly encourage questions in the class. And I do thank students for asking questions so that we learn together. So to me, the class is more effective when it’s not just teacher talk and where there are ways intentionally built in for students to share their responses.”

The teachers also saw the importance of getting their students to be motivated about learning. They believed that a motivated student will concomitantly produce better work quality and have less disciplinary issues. Teacher S2T9 (male) expressed his views, “…the whole idea is for the students to understand why they are learning what they are learning, rather than getting them to learn just the content itself. I think the motivation to understand why it is that they are learning a particular concept, or a particular set of content is more important to me, because in the end, the motivation will be intrinsic, rather than me forcing it down on them.” Teacher S16T108 (male) was of the view that when “my students are motivated, there will be less problems in class as well. When it comes to work quality and disciplinary issues, I think it is better.” He also thought that, “…this is a long-term solution in making them do well in class, instead of going on the hard way of discipline. I think this is more sustainable…it’s better than to scold every day.”

Ultimately, the teachers aimed to nurture autonomous learners. They concurred with the SDT’s philosophy in supporting the students in owning their learning through according them greater autonomy in the learning processes. Teacher S8T56 (male)‘s articulation helped us understand some of the teachers’ psyche, “I feel that giving the ownership back to them, giving them the freedom to do certain things, may allow them to get more interested in the subject, allow them to work even harder, clarify their doubts, be more curious or inquisitive in asking questions.” And this belief drove their pedagogy to teaching and learning as communicated by teacher S5T33 (female), “Because I believe that learning should not be a top-down approach. They are not sponges so they should have a say in certain things they learn…So at the start of the semester or year, I will ask them, ‘What kind of learning would you like to see your classroom?’…I will try to cater to that as much as possible.”

One other reason that surfaced from the interviews was the teachers’ desire to improve their relationships with their students (5 of 59 teachers), which they believed would better rapport and allow them to connect with their students better. On nurturing relationships, Teacher S13T87 (female) remarked, “I believe that for human beings, the relationships, the interactions, are the most important…when you have that relationship and the understanding, things will be more effective.” She continued, “Once the teacher-student relationship is built up and is strong, they will feel safer to learn in class. They will also be more willing to learn and to try…they have experienced different forms of failure…For many of them, their belief is ‘I cannot’. So, I think when they feel respected, they feel that they are given the opportunity to learn what they want to learn and given the opportunity to ask questions, it means quite a bit to them.” On the alignment between autonomy-supportive teaching and her teaching philosophy on nurturing positive relationship, teacher S1T4 (female) opined, “this feels like a more respectful approach, from teacher to student, and it generally creates a respectful environment. It shows that the students’ thoughts are valued − the part where we take the students’ perspective…Students feel they are part of the learning process, not just me telling them what they have to do. I feel these are the two main advantages − respect and getting students more involved.”

In nurturing positive relationships, the teachers were by extension, creating a positive classroom culture. In wanting to create a positive classroom climate, they were guided by the beliefs that a “safe and happy environment” (S16T108, male) is most conducive for students’ motivation and learning. Teacher S11T73 (male) elaborated, “A positive classroom culture is definitely one which I think is very helpful and very important…being in an environment where the students can make mistakes and dare to voice their thoughts, I think students can only do that if they are comfortable with their classroom environment…I think that it (autonomy-supportive teaching) lands itself very well into building this type of positive classroom culture that would provide a safe environment for these things to grow and happen.” Teacher S16T106 (male) agreed with the above-mentioned view. He said, “What I’m concerned about is that during my lessons, my students enjoy the subject, feel safe in asking or approach me for help…That shapes my philosophy of being there for them.”

Contrasting with the above-mentioned philosophy are two other sets of teaching philosophy on discipline and collectivism, which we will discuss in turn. This teaching philosophy on discipline stemmed from the belief that there is a need to be hardy and tough in order to survive and thrive in this competitive world that we live in. The following excerpt excellently captures such a philosophy and belief.

Teacher S5T31 (male) said, “Prior to this, I believe in the word ‘discipline’. I’m more of the discipline type.” In his communication of his philosophy on “discipline,” the teacher also conveyed his belief on developing a generation of people who are resilient. Explicitly, he elaborated, “In the past two years due to COVID, the school tried to help (minimise stress) by reducing some of the examinable topics and even giving them some leeway to extra time. But at the end of the day, they will still grow up into the working world and in time to come, people will forget that there’s such a thing (COVID). Let us face it, we had SARS 17 years ago. That was when I graduated. By now, you might have realised that none of the students knew what had happened. But when you go out to work, people are more concerned about your ability. As much as the school management says to allow the students to grow their own self-confidence, but will the students grow stronger from this?” Because of his teaching philosophy and belief, he was less receptive to the autonomy-supportive approach as he was afraid that if he “turned softer” meaning enforced less discipline, he would “lose my students” in that “since the Math teacher will not punish me, I will not do his work.” (S5T31, male) Teacher S4T25 (male) presented another reason on why teachers in the discipline camp tended to be more controlling and less receptive to being autonomy-supportive. He confessed, “I am of the controlling type. Because to me, having a sense of self-discipline is very important to students’ building their own understanding of themselves, and being able to do *what it takes to do*, rather than do what they like to do…It is important to have discipline…” And thus, teachers in the discipline camp would choose to practise “tough love” (S4T23, female) over being overtly autonomy-supportive.

Another philosophy focuses on collectivism and the belief of others before self. Teacher S17T111 (male) thought aloud his concern, “When we try to be very student-centric, the students have become more self-centric, because everything is about me, even in the family and school. When we try to be more student-centric, we give them choices, we try to cater to them, we try to relate to them, everything is for them. So sometimes the students may have a feeling of entitlement, ‘I’m entitled to this, you must do this for me, you must give me choices, if you do not give me choices, I will not respond’…The system must work for them, the teachers must support them, must put them in the centre and provide the layers of support for them.” For teachers in this camp, they felt that whilst educators strive to nurture students’ intrinsic motivation by supporting their autonomy, there is also a need to balance the practices.

### 3.2. Personal experiences

The teachers’ personal experiences (32 of 59 teachers) which refer to their personal life encounters both as students themselves and now as teachers, also influenced their motivating style.

Their experiences as students at the receiving end taught them how they preferred to be treated as students and with this insight, they formulated their personal motivating style. Teacher S11T76 (female) succinctly expressed this point, “I feel that a large part is dependent on our own education process, what we ourselves have experienced as students.” Teacher S1T1 (female) reflected on her childhood experience, “It stems from my experience. I do not like being scolded. If anyone felt that I was doing something wrong and they explained to me, I would understand and I would think about it and I would change if I think I need to. And I feel that I would want to extend that courtesy to anyone be it whether they are 13 or however old they are. I believe that we can treat each other with respect, we can be kind.” Teacher S13T87 (female) detailed her experience to show how powerful and memorable the teachers’ words and behaviours could be on a child, “When I was young, I asked a question at the beginning of the year…and the reaction of the teacher was, ‘Don’t have common sense?’… I think how we respond, how our body language is, how our tone is, the words we use, we have to be very careful…they may be younger than us, but I think the respect accorded should be accorded to any human being.”

Whilst their experiences as students taught them about the lasting impact of a teacher’s words and deeds on a student’s psyche, their experiences as teachers showed them that the strategies could help them build rapport, earn respect, and connect better with their students. With a better relationship with their students, they in turn felt energised and more encouraged to be autonomy-supportive with their students. Teacher S17T111 (male) articulated on his observation of the changing educational landscape and his view on autonomy-supportive motivating style as the more adaptive teaching style going forward. He said, “The profile of the students changes…in the later years of my teaching, I find that the way I relate to students cannot be the same as in the past where we used more controlling method. I realise I need to connect with them more…they are more well read, more IT-savvy, and they are faster than us in terms of processing…So I started to change. I started to give students more autonomy. I wanted to hear more from them. I gave them choices.”

Adding to the conversation, Teacher S1T4 (female) shared her learnings accumulated over the years, “I have come to understand that emotions play a role in the way people think and do things…I think bringing the environment to a positive state would help their emotions, if they are feeling bad for that day.”

Comparing autonomy-supportive versus controlling teaching style, another teacher gave her take on what influenced her motivating style. She narrated, “When I started off as a beginning teacher (BT), my mentor happened to be someone with a controlling style and as a BT, I thought to myself, ‘Maybe teachers are always like that. There’s only one style.’… In my first year, I found myself doing controlling teaching. I realised that it did not derive any benefits besides getting students to be fixated or controlled into doing certain things… and I found myself losing students along the way. For example, I could see the students not approaching me to ask questions. They were just going with their peer groups to figure out solutions and they did not want to seek help from the teacher… So when I found out that this controlling behaviour had these repercussions on the students, I felt I needed to change. At that point in time, I also saw how a few of my colleagues with controlling behaviours affected their students emotionally. This was how I changed to the autonomy– supportive behaviours. I started to taste success in it as I went on to teach upper secondary the next year. I found that this suits upper secondary (students) very well…” (S13IDT89, female).

And with the implementation of autonomy-supportive teaching, the teachers began to experience better rapport and interaction, and mutual respect between themselves and their students and these positive mutualistic experiences spurred them to continue with being autonomy-supportive. Teacher S14T96 (male) shared about his experience, “I feel that was respecting the students more. And they also felt that I was trying my best to respect them, so it helped build a stronger rapport in the class.” With the rapport, the students had greater respect for the teachers, and they were more willing to learn.

Teacher S7T47 (female) articulated, “I spoke to these four musketeers (rebellious and challenging students in the class) and asked, ‘why are you afraid of Ms. X?’ They replied, ‘What afraid? We respect her, okay. We are not afraid.’…Then I asked, “What makes you respect her?” They replied, ‘She is firm, but she understands, she will listen to us.’…I told myself ‘I want my students to be respectful of me. I do not want them to be afraid of me.’ …that is how it made the style I have today.” The same teacher reiterated, “I believe in building good rapport…The first step to building good rapport is to make them not afraid of me. When they are not afraid of me, they are more willing to listen to what I’m teaching.”

And when the teachers experienced success in their interactions with their students, they were more encouraged to continue with the autonomy-supportive teaching. Teacher S14T97 (male) provided his takeaway from his experimentation, “I would say definitely is the satisfaction, the interaction I get from my students, being able to interact with them more, being able to get direct feedback from them more. It definitely has helped me in terms of structuring my teaching, helped me in terms of wanting to cater and structure the lessons to suit their needs.” This experience was shared by Teacher S3T18 (female) who said, “it’s really good that you know how this whole thing has changed my mindset? I mean to be very honest when I attended the workshop, I did not really pick up a lot of strategies in that sense because the strategies were a little hard for me to understand how I could apply them into my subject area. But I think trying out the big rocks like they said getting their (the students’) perspectives, see if they enjoyed the activities, just starting small gave me the confidence to do it further.”

Interestingly, some teachers felt themselves grow from the experience, became transformed and spoke of their intention to continue being autonomy-supportive inside and outside the classrooms. Teacher S15T102 (male) shared, “this study has made me more conscious of how students react to the way I carry out my lessons, how I emphasise certain key points, how I communicate with them…having participated in this study, sometimes when you keep making good effort in trying to change the way you do certain things, it becomes a habit? In some ways, my experience has made me extend beyond the study…” Indeed, as the saying goes “Success begets success.”

### 3.3. Motivation to teach

The teachers’ motivation to teach (14 of 59 teachers) which refers to their intrinsic drives for teaching was another factor that influenced their employment of autonomy-supportive strategies. For the teachers who were eager to try out the strategies, they were convinced of the theoretical underpinnings of the SDT, were keen to nurture their students’ intrinsic motivation in learning and to improve their students’ learning outcomes such as having greater self-confidence and self-esteem, greater willingness to explore and put in effort in learning, and to enjoy learning. Some teachers also saw the autonomy-supportive strategies as useful techniques for them to reach out to and connect with their students.

Teacher S11T73 (male) expressed his motivation succinctly, “I wanted to get them (the students) more motivated – intrinsically motivated. I wanted to see the results of it.” Teacher S14T96 (male) elaborated, “…I figured along the way what matters more was how they feel about their learning experiences…if you do not hit at the students’ hearts, you cannot get them. So, the intrinsic motivation hits them at the sweet spots, the hearts of the students. I think it will make learning more meaningful for them.” Teacher S11T76 (female) summed up the teachers’ motivation in wanting to nurture intrinsic motivation, “I believe it (learning) should come from within them, wanting to do well.”

Further on the teachers’ motivation to teach, Teacher S6T42 (female) spoke of her wish which was to “…get the students to build their self-esteem. And hopefully they find that they are capable of doing more than what they thought they can do.” Teacher S17T115 (female) wanted her students “to be willing to do the things in class, like more willingly, if they know the rationale behind why certain things are to be done in a certain way…” Teacher S3T19 (female) was motivated to give her students a positive learning experience. She shared, “I want the students to enjoy my lessons. I want them to explore and learn things that’s not in the textbook…I enjoy teaching in this way, and I’ll enjoy it if I’m the students.” Teacher S2T12 (female) elaborated, “…it is to build a mutualistic relationship between the teacher and students because it benefits both…I would love to have an environment where both myself and my students feel we are both in sync, we are both contributing to the learning and we are part of the learning community.”

Some teachers also saw the autonomy-supportive instructional behaviours as very suitable strategies to reach out to and build relationships with their students. Teacher S17T111 (male) surmised, “…my motivation is, I’ll be able to reach out to my students more extensively.” Thus, the teachers were motivated to use the autonomy-supportive approach in their classrooms.

### 3.4. Personality

The teachers’ employment of autonomy-supportive instructional behaviours was influenced in a large part by the teachers’ personalities (13 of 59 teachers) which refer to their innate traits. For the teachers who were more empathetic and nurturing to begin with, they were more inclined towards the autonomy-supportive teaching style. For teachers who were less compromising and stricter, they were less likely to adopt the autonomy-supportive teaching style; and if they did attempt to try out the strategies, were less likely to persist in situations of time urgency and stress. The extracts below presented the teachers’ self-admitted personal dispositions and their self-reflections with regard to their motivating styles.

One teacher who proclaimed to have a more nurturing personality shared, “After learning about what autonomy-support means…I find that it aligns quite closely with what I usually do in class…I did not have to intentionally or purposefully make drastic changes to my teaching style. I did not have to make a big deviation from what I usually do in class.” (S17T111, male) Because the teachers’ predisposition was towards autonomy-supportive teaching, the recommended autonomy-supportive strategies were not demanding of them. The teachers presented a contrast to help us understand what could have been challenging for them. One teacher offered an explanation, “…some teachers are very fierce. But for me if I want to be very fierce, that means I’m putting extra effort.” (S8T55, female) The above point was elaborated by another teacher, “If I try to be the opposite which is controlling and authoritarian, it’s me trying to be someone who is not like me. So, it drains me in this case.” (S16T106, male).

For teachers who admitted leaning towards the controlling end, although they had made some attempts at being autonomy-supportive, they found implementing the strategies effortful. The following conversations help us understand the teachers’ struggles and the psychology behind the teachers’ teaching style. Teacher S5T31 (male) said, “I’m more of the discipline type…the biggest challenge for me is that it’s not my style. It’s not possible that after the course, I will immediately become like that (autonomy-supportive). It does not work that way…with the exams round the corner, can I give them so much autonomy? Am I not worried about how they are going to fare? It’s some sort of a torture. I am quite torn between these two things (being autonomy-supportive and being controlling).” Teacher S1T2 (male) articulated, “…trying to do the switch from controlling to autonomy-supportive (style) is really one of the biggest challenges. It really takes a lot of courage to use a different tone, a different kind of non-pressuring language. It can sometimes be quite challenging, especially if we have been teaching for so many years and all this while we have been controlling. Suddenly we have to change our style, it’s not easy.” Teacher S17T114 (female) offered her insight on her colleagues’ perceived difficulty in implementing the autonomy-supportive strategies. She said, “it’s not about their comfort level, but probably a habit and a style that they have developed over the years…if we want teachers with certain years of experience to change their style of teaching, I think it’s a little bit harder for them to adapt. So, it’s probably not because they do not want to try it out, it’s a habit and it’s in their habit to carry out a certain style of teaching.”

Indeed, an ingrained disposition and entrenched habitual tendency cannot be changed overnight, but we believe that with intentional, conscious effort and persistent practices, old habits can be changed, and new habits can become part of one’s nature and character. We were thus quite encouraged when some of the teachers who cognitively believed in the SDT underpinnings and practices were adamant in persisting with their effort. Teacher S11T76 (female) spoke of her resolution, “…I will still continue to apply the strategies. It takes time. It has to become a nature. I mean I must be able to use it naturally. It has to be part of my repertoire and it’s something that should come naturally. So right now, I’m still trying to apply it very intentionally such that eventually it becomes part of myself.” We agree with the teacher that to be authentically autonomy-supportive, embodiment is key.

### 3.5. Teachers’ mental and emotional states

Next, being human, the mental and emotional states of the teachers (5 of 59 teachers) which refer to the states of mind of the teachers, could influence their abilities to carry out autonomy-supportive teaching. According to the teachers, implementing autonomy-supportive instructional behaviours required conscious effort and a mental and emotional state of calm could facilitate the execution of the intentional effort. However, when they were overwhelmed by stress, were low in energy or were not feeling well, they found it difficult to sustain the effort.

Substantiating the above statement, Teacher S4T25 (male) stated, “…because I’m more calm, it becomes easier for me to give them praises.” In contrast, emotions can affect the teachers’ abilities to implement the autonomy-supportive strategies. Teacher S15T99 (female) shared her internal struggles, “…it’s my own mental state. If I’m stressed, I would not have the capacity to think of all these acts…because doing all these acts take effort.” Teacher S16T106 (male) added, “…everyone is human, so on some days when you feel that you are very low on energy, it’s really up to you on whether you want to put in the extra effort to be motivating.” Teacher S14T96 (male) shared a similar sentiment, “…there are days when I am really tired, I’m not feeling well. So, for those days, to be honest, because we do have very bad days, I did have some problems. I planned for it but my state of mind was not ready. For example, I was struggling in my job, I planned for it (to be autonomy-supportive), but I’m upset, I cannot do it 100 percent because I’m not 100 percent ready.” To sum up this section, the mental and emotional states of the teachers is one factor that can influence their implementation of autonomy-supportive teaching.

### 3.6. Teacher efficacy

The last factor that influenced the teachers’ employment of autonomy-supportive strategies was their sense of teaching efficacy (2 of 59 teachers) which refers to their felt sense of competence in teaching. If they did not feel confident about the subject content, they were less likely to modify their tried-and-tested teaching method to experiment with new pedagogical moves.

Teacher S16T106 (male) confided his concern, “I would say is the subject that I have to teach. I do not feel as competent in teaching my second teaching subject as compared to my first…The fact that I’m given a graduating class adds on to my stress level because I understand the stakes that I have on hand.” And the teachers would be more willing to implement autonomy-supportive teaching if they had more knowledge of the strategies and felt more confident in translating those strategies into classroom practices. Teacher S3T17 (male) confessed, “I’ve not known enough, how to translate the autonomy-supportive strategies…the doing part. If there are other examples, other than the ones I have tried, maybe I will try.”

## 4. Discussion

Reeve (2009) professed that awareness is a powerful mean to changing one’s behaviours. Specifically, when one has awareness of, that is, has identified, understood, and attended to the internal factors that push and pull one’s behaviours − either consciously or unconsciously, intentionally or unintentionally, one can then be more mindful of the drivers that direct one’s behaviours. In the context of our study, a teacher’s greater awareness of the personal factors that induce him or her towards a controlling motivating style and the inimical consequences the controlling instructional behaviours would have on their students in the long run, presents an opportunity for the teacher to reflect on and to make a conscious choice towards his or her teaching behaviours. As the teacher becomes more mindful of the causes and consequences of his/her motivating style, he/she gains a greater capacity to make deliberate choices on his/her behaviours – either to behave in a flexible, autonomous, and adaptive way, or to surrender to habitual and oftentimes reactive teaching behaviours. With this greater awareness of the drivers that facilitate or frustrate him or her towards supporting his/her students’ autonomy, the teacher can then make a conscious choice to teach in a more autonomy-supportive way (Brown and Ryan, 2003; Ryan and Deci, 2004; Brown et al., 2007; Reeve, 2009; Rigby et al., 2014; Schultz and Ryan, 2015). Thus, identifying the personal factors that influence the teachers’ motivating style as this study has done has its implication value. The awareness accorded by the information provided is a key first step in the effort to empowering teachers to become less controlling and more autonomy-supportive.

From the interviews, we identified “teaching philosophies and beliefs” (49 of 59 teachers), “personal experiences” (32 of 59 teachers), “motivation to teach” (14 of 59 teachers), “personality” (13 of 59 teachers), the teachers’ “mental and emotional states” (5 of 59 teachers), and “teaching efficacy” (2 of 59 teachers) as the personal factors influencing teachers’ motivating styles.

First and foremost, the teachers’ teaching philosophies and beliefs were the most important influences of their teaching practices. If they embraced the teaching philosophies of nurturing the child, the relationship, and the environment, they were more likely to adopt autonomy-supportive teaching; and if they strongly believed in discipline and collectivism over individualism, they were more likely to employ controlling teaching. The teaching philosophy of nurturing the child, the relationship, and the environment stemmed from the underlying belief that “every child matters.” In nurturing the child, the teachers wanted to foster the love and curiosity for learning. Regarding relationship, they desired to make the students feel cared for and respected. In supporting the environment, they aimed to create a safe place for the students to explore, learn and grow. Contrasting with the above-mentioned teaching philosophy was another set of philosophy on tough love. Undergirded this belief was a mindset that we live in a harsh and competitive world, things do not always happen the way we want them to, and we have to be adaptive and tough in order to survive. The teachers who held this belief were also likely to have this view that they might be taken advantage of if they adopted the softer autonomy-supportive approach. Additionally, some other teachers brought up in a collectivist society such as Singapore were concerned that developing the individual’s self-determination would make the students too individualistic and for this, they were of two minds about supporting the students’ autonomy. Indeed, teachers’ beliefs were strong driving forces of their behaviours (Reeve et al., 2014; Reeve and Cheon, 2014). Reeve et al. (2014) had earlier reported that when teachers believed that autonomy-supportive strategies were culturally normal and effective, they were more willing to implement the motivating style. However, in many educational systems around the world particularly in collectivistic societies such as Singapore, controlling motivating style may be a more culturally valued teaching style (Reeve, 2009; Reeve et al., 2014). Many teachers view controlling strategies as the more efficient and effective way to motivate students to produce maximal performance (Reeve, 2009). These views may be reinforced by educational policies that are pressuring which further entrench such controlling teaching practices (Maehr and Midgley, 1991; Reeve, 2009). SDT scholars argued that whilst controlling strategies might yield immediate benefits (for example, situationally turning motivation “on”), these controlling practices overlook the less salient long-term negative effects these strategies might have (for example, developmentally turning motivation “off”) on the students’ learning. They proposed autonomy-supportive strategies as the solution to long-term self-regulation and learning (Vansteenkiste et al., 2005; Reeve, 2009).

The second most mentioned factor was the teachers’ personal experiences – both as a student and a teacher. Their experiences when they were students taught them what and how they preferred to be treated which guided their teaching practices; and their experiences with autonomy-supportive teaching showed them that this teaching method can help them connect better with their students which further encouraged them to continue with this teaching approach. Adding to the above-mentioned point, the teachers in Parr et al. (2021) had also mentioned negative experiences with their own teachers as good learning points for them on what not to do and what they could have done differently to make a more positive impact on their students’ lives.

The third personal factor was the teachers’ personal motivation to teach. The teachers were intrinsically motivated to support their students’ growth and learning. They were persuaded by the tenets of the SDT and agreed with the SDT’s proposal on the importance of nurturing students’ autonomy so that the students would own their learning. Hence, they were very willing to experiment with the autonomy-supportive style. Our finding is consistent with existing research work (Pelletier et al., 2002; Taylor et al., 2008; Hein et al., 2012) which showed that teachers with autonomous motivation to teach (desiring to make a difference in their students’ lives and in their community) were found to be more autonomy-supportive by centring their instructions on students’ needs through the provision of choice, incorporation of students’ voices, and making lesson activities meaningful for their students. In contrast, when the teachers’ own motivation to teach was characterised by non-autonomous motivation, they tended to motivate their students in controlling ways (Cai et al., 2002; Pelletier et al., 2002). Taylor et al. (2008) explained that the teachers’ autonomous motivation to teach could in part be due to their psychological needs being met in their schools. For these teachers, they were very likely to have autonomy in deciding how they wanted to deliver their lessons, to feel competent in their teaching and could relate with their colleagues and students, and in turn more likely to create the same learning environment for their students.

The fourth factor was the teachers’ personalities. For the teachers who were more nurturing to begin with, they were more inclined towards the autonomy-supportive teaching style as it was in their nature to be supportive; and for teachers who were less accommodating, they were less likely to adopt the autonomy-supportive teaching style as they required extra effort in supporting their students’ autonomy. For these “controlling” teachers whose “controlling” strategies were tried, tested, and proven to work for them, getting out of their comfort zone to experiment with an alternative and unfamiliar method required much courage from them. Previous studies had also reported that personal dispositions (autonomous causality orientation, self-compassion) predicted teachers’ use of motivational strategies (Deci and Ryan, 1985; Taylor et al., 2008; Moè and Katz, 2020). Reeve (2009) elaborated that the teachers’ prior beliefs and dispositions influence how new information is attended to, processed and eventually whether it is accepted or rejected. More specifically, when teachers harbour autonomy-oriented beliefs, motivations, values, and personality dispositions, they tend to embrace practices on supporting students’ autonomy with acceptance, assimilation, and self-integration. However, when the above-mentioned personal attributes are oriented by control, they would react to these same practices with scepticism and resistance – being autonomy-supportive is not in their mental and behavioural schemata (Reeve, 1998). This, however, does not mean that teachers with entrenched controlling style cannot learn to be autonomy-supportive. According to Reeve (2009), information about autonomy-support and its classroom practice needs to be presented to control-oriented teachers in such a way that it creates a sense of dissatisfaction with their current controlling approach to motivating students. It is under these conditions that the control-oriented teachers are more likely to try out alternatives to their long-held approach.

As the fifth factor, the mental and emotional states of the teachers influenced their abilities to carry out autonomy-supportive teaching. When they were overwhelmed by stress, were low in energy or were not feeling well, they found it difficult to persist with autonomy-supportive teaching because such a teaching method required the teachers to be mentally and emotionally present for their students, a finding that echoed that of previous research work such as Taylor et al. (2008, 2009). Bearing in mind that the current study was conducted during the COVID years, it is a credit to the teachers that they were still willing to learn and try to be autonomy-supportive.

Finally, the teachers’ felt sense of competence in teaching the subject influenced their ability to employ autonomy-supportive teaching. Specifically, if the teachers were very confident about teaching the subject, they were more willing to play around with their teaching methods. Additionally, earlier studies (Leroy et al., 2007; Bennett et al., 2017; Parr et al., 2021) had reported that the more the teachers felt confident in helping their students overcome difficulties in schools, the more they employed autonomy-supportive strategies. Thus, teachers’ felt sense of teaching efficacy can influence their willingness and ability in trying out novel ways of teaching. Identifying this factor is useful. If we can create conditions favourable to supporting teachers’ teaching efficacy, it could in turn allow them to create classroom climates that support their students’ autonomy.

## 5. Conclusion

The findings of this study have practical implications for the individual teachers and the social environment that supports them.

For the individual teachers, by articulating and understanding the reasons why they or their colleagues adopted and implemented the autonomy-supportive instructional behaviours or not, they brought to conscious awareness the implicit and explicit processes that pull and push their daily behaviours. This could contribute to an awakening which could in turn, help them to reflect on what they have said or done in their classrooms and to intentionally modify their classroom behaviours. By foregrounding the personal factors that can foster or frustrate the teachers’ expression of autonomy-supportive instructional behaviours, this paper hopes to contribute to such an awakening.

For the social environment (policy makers and school leaders) that supports the teachers, having a good understanding of the teachers’ “within” reasons influencing the expression of autonomy-supportive teaching may allow them to better support the teachers. For example, in understanding that the teachers’ entrenched “teaching philosophies and beliefs,” “personal experiences,” “motivation to teach” and “personality” are hindering the expression of autonomy-supportive teaching, policy makers and school leaders can then take a more persuasive approach in encouraging the teachers to be autonomy-supportive. One way to do so is to create a dissonance between the teachers’ current teaching approach and their vision in nurturing motivated and self-regulated lifelong learners, which Reeve (2009) suggested may persuade the teachers to reflect and alter their teaching behaviours. For teachers who are stressed and who do not feel competent in implementing the autonomy-supportive strategies, schools can provide them with the emotional support and ideas on teaching strategies to help them better motivate their students.

One limitation pertains to the use of qualitative interviews which presented a challenge of possible social desirable bias. This challenge had been anticipated and the teachers were assured of confidentiality in their responses. Nonetheless, we are cognisant that the phenomenon could not be eradicated totally. As another limitation, this study focussed only on the perspectives of the teachers. It would be useful to triangulate the teachers’ perspectives with the students’ views on their teachers’ personal factors, their observations of their teachers’ teaching styles, and how these teaching behaviours affect their learning such as their motivation to learn and academic performance. Such triangulation of views would give greater credibility to the teachers’ self-reported behaviours. Any discrepancy in views could also provide the impetus for teachers’ self-reflections and researchers’ further investigations.

For recommendation, previous studies (Pelletier et al., 2002; Leroy et al., 2007; Taylor et al., 2008, 2009; Reeve, 2013; Rocchi et al., 2013; Hornstra et al., 2015) had suggested that contextual factors such as school-related factors and student-related factors can influence the teachers’ motivating styles. There may also be gender difference in the adoption and implementation of the motivating styles (Cai et al., 2002). The age of the teachers and number of years of teaching experience may also matter in the teachers’ willingness and ableness in adopting autonomy-supportive motivating style. Future studies can explore the contextual factors, gender, age and/or number of years of teaching experience so that we can have a more comprehensive understanding of the factors that may influence the teachers’ motivating styles; which would be the basis for better classroom climates that could improve the students’ motivation and learning outcomes.

## Data availability statement

The original contributions presented in the study are included in the article/supplementary material, further inquiries can be directed to the corresponding author.

## Ethics statement

The studies involving human participants were reviewed and approved by Nanyang Technological University Institutional Review Board (IRB-2021-03-033 and IRB-2021-03-033-01). The participants provided their written informed consent to participate in this study.

## Author contributions

WL, CW, YK, BN, KL, and JR: conceptualised the study. LK, YK, and BN collected and analysed the data. LK and WL wrote the paper. All authors contributed to the article and approved the submitted version.

## Funding

This project was supported by the Education Research Funding Programme funded by the Ministry of Education, Singapore (OER 12/19 LWC).

## Conflict of interest

The authors declare that the research was conducted in the absence of any commercial or financial relationships that could be construed as a potential conflict of interest.

## Publisher’s note

All claims expressed in this article are solely those of the authors and do not necessarily represent those of their affiliated organizations, or those of the publisher, the editors and the reviewers. Any product that may be evaluated in this article, or claim that may be made by its manufacturer, is not guaranteed or endorsed by the publisher.

## Annex A

**Interview questions**

First, we would like to know if you have continued with being autonomy– supportive throughout the semester?

If no,

What happened? What were the challenges?

Was there a time you were motivated/not motivated to be autonomy-supportive? What happened?

What are the personal reasons/factors that discouraged you from adopting the autonomy-supportive motivating style?

If yes,

What was your motivation to be autonomy-supportive in your lessons?

Was there a time you were motivated/not motivated to be autonomy-supportive? What happened?

Were there any personal reasons/factors that encouraged or discouraged you from adopting the autonomy-supportive motivating style?
